# “Seek and find” or “search and destroy?” Identifying and retaining “healthy” stool donors for fecal microbiota transplantation

**DOI:** 10.1002/jgh3.12892

**Published:** 2023-03-23

**Authors:** Cyriac Abby Philips

**Affiliations:** ^1^ Clinical and Translational Hepatology and Monarch Liver Laboratory The Liver Institute, Center of Excellence in Gastrointestinal Sciences, Rajagiri Hospital Aluva (Kochi) India

Fecal microbiota transplantation (FMT) has come a long way from its crude descriptions as “yellow soup” or “golden juice” in traditional Chinese medicine texts dating back to 770 BCE.^1^ Classical therapeutic handbooks of traditional Chinese medicine compiled by “monk‐physicians” during various dynasty rules contained details on versions of human and nonhuman fecal preparations – from freshly made suspensions to fermented broths and dried powder forms used for treating various illnesses, predominantly food poisoning and fever.^2^ From surgeon Ben Eiseman's FMT enema for patients with “pseudomembranous colitis” in 1958 to the Food and Drug Administration's approval of the FMT‐based product, Rebyota, to prevent the recurrence of *Clostridioides difficile* infection in individuals 18 years of age and older, it would seem that the therapy of the future is making slow but long strides.^3^, ^4^ Central to an FMT program is the stool donor, who is difficult to recruit and retain. In the patient‐selected donor model, a substantial logistical burden falls on the physician to complete screening on time. This could result in protocol lapse or arbitrary changes to screening tests that may be driven by the physician's “humane” bias toward patient‐related factors such as screening costs, relationship to donors, and pressure to commence treatment. Using FMT from stool banks negates these logistical concerns and should ideally become the universal standard.^5^ Using universal rather than patient‐directed donors results in significant cost savings and timing efficacy. However, a rigorous screening process eliminates most potential donors given a history of allergies, asthma, gastrointestinal disorders including functional bowel disease, autoimmune diseases, high prevalence of metabolic syndrome, and recent travel or use of antimicrobials in the previous 3 months. Countries have reported variable donor pass rates after exhaustive screening protocols within their stool banking programs, from 0.6 to 0.8% in Toronto and Hong Kong to 25–30% in Italy and the United States, respectively.^6^ Donor retention rates further reduce as a result of a lack of interest in long‐term donation, intercurrent infections, use of antimicrobials, and travel. Even though these shortcomings and difficulties are universally faced by stool bank programs, a small but steady donation state has helped maintain the demand–supply chain of FMT for therapeutic and in clinical research protocols.

In this context, the study by Emily Tucker and colleagues in *JGHOPEN* adds to our current knowledge of stool‐banking‐related demand–supply outcomes and the struggles in identifying healthy stool donors and their retention inside the program.^7^ In their study, only a minority of potential stool donors were eligible to enter BiomeBank's donor program. Nearly 60% were excluded from answering a written questionnaire, only 8% proceeded to clinical assessment, and, finally, 4.5% were enrolled in the program. Only 50% of enrolled donors could be retained. All donors were ineligible to donate at some point, and asymptomatic donors were also found to harbor extended‐spectrum beta‐lactamase‐producing Enterobacterales species. Robust but justifiable screening of interested stool donors narrows the potential for donor enrolment in FMT programs. *Re*‐qualification of donors, including completing a written questionnaire, clinical assessment, and visual inspection of donated stool, during repeat donation results in further donor attrition. In programs that also include molecular analysis of stool at each donation, the chances of destroying processed samples in the quarantine period seem high, leading to a shortage of stored, usable samples.^8^ As the authors rightly point out, a major limitation in their program was the lack of “donor comfort” for providing samples. Donors had to travel to a Good Manufacturing Practice‐licensed facility for on‐site defecation and repeatedly reveal personal information that would rescind their participation. Even with such challenging situations, the BiomeBank could robustly screen, timely store, effectively supply, and satisfactorily meet demands from clinical hospitals and research facilities catering to FMT.

Nonetheless, more than squeezed in contentment, stool banking programs must aim to increase supply over and above demands so that more clinical facilities can include FMT in their routine practice as recommended treatments or within research settings. This could be made possible by approaching the screening program pragmatically. We must welcome the fact that there can be no internationally acceptable blanket guidelines on donor screening that stool banks across the globe can follow due to the wide cultural, social, dietary, genetic, and economic variations and heterogeneity in the prevalence of communicable and noncommunicable diseases within different communities that remain unmodifiable. For instance, in a Dutch cohort, the presence of the protozoa *Dientamoeba fragilis* and *Blastocystis* species were frequent reasons for the ineligibility of potential donors.^9^ Such reasons for donor exclusion are yet to be reported from other large stool‐bank‐based recruitment analysis studies. In this context, screening guidelines must be “regionally driven” to ensure a logical process that satisfactorily addresses safe donation and donor acceptance of the long‐term donation. Public awareness on the importance of stool banks as an emerging therapeutic armamentarium akin to blood banks still needs to be met. Expanding collection centers strategically placed across major cities could help improve the acceptance of sustained donations and address donor tolerance and comfort. Stool specimen collection in a clinical setting or at a predefined facility may not be feasible for most persons who wish to enroll in the program. The timing of bowel evacuation is highly diverse between persons, genders, ages, and among different communities, depending on socioeconomic, psychosocial, environmental, dietary, and occupational factors.^10^ Including easy‐to‐use customized stool collection kits for home collection could further help improve enrolment and sustained stool donation.

At the end of the day, the process of stool banking and its maintenance is a costly affair. The burden to improve public health and to maintain the right to health for all when it comes to the inclusion of exceptional, rare, and emerging therapeutic options such as FMT should rest on the shoulders of both public and private healthcare sectors. Even though infrastructural capacity is notable, the difficulty of sustained funding for running and maintaining stool banking facilities precludes the participation of the private healthcare sector in establishing stool banks. Reasonable and justifiable participation from both private and public health sectors can ameliorate this discrepancy so that stool banks can be established and made operational with the pertinent resources available from both parties. In developing countries without a centralized healthcare system, the participation of the public sector gives credibility to the program and improves funding options, and that of the private sector helps promote state‐of‐the‐art infrastructural capabilities. This would ensure a supportive regulatory framework, affordable and quality‐assured FMT, sustained funding, and options for reimbursement, which are essential in maintaining stool banks.

**Figure 1:**
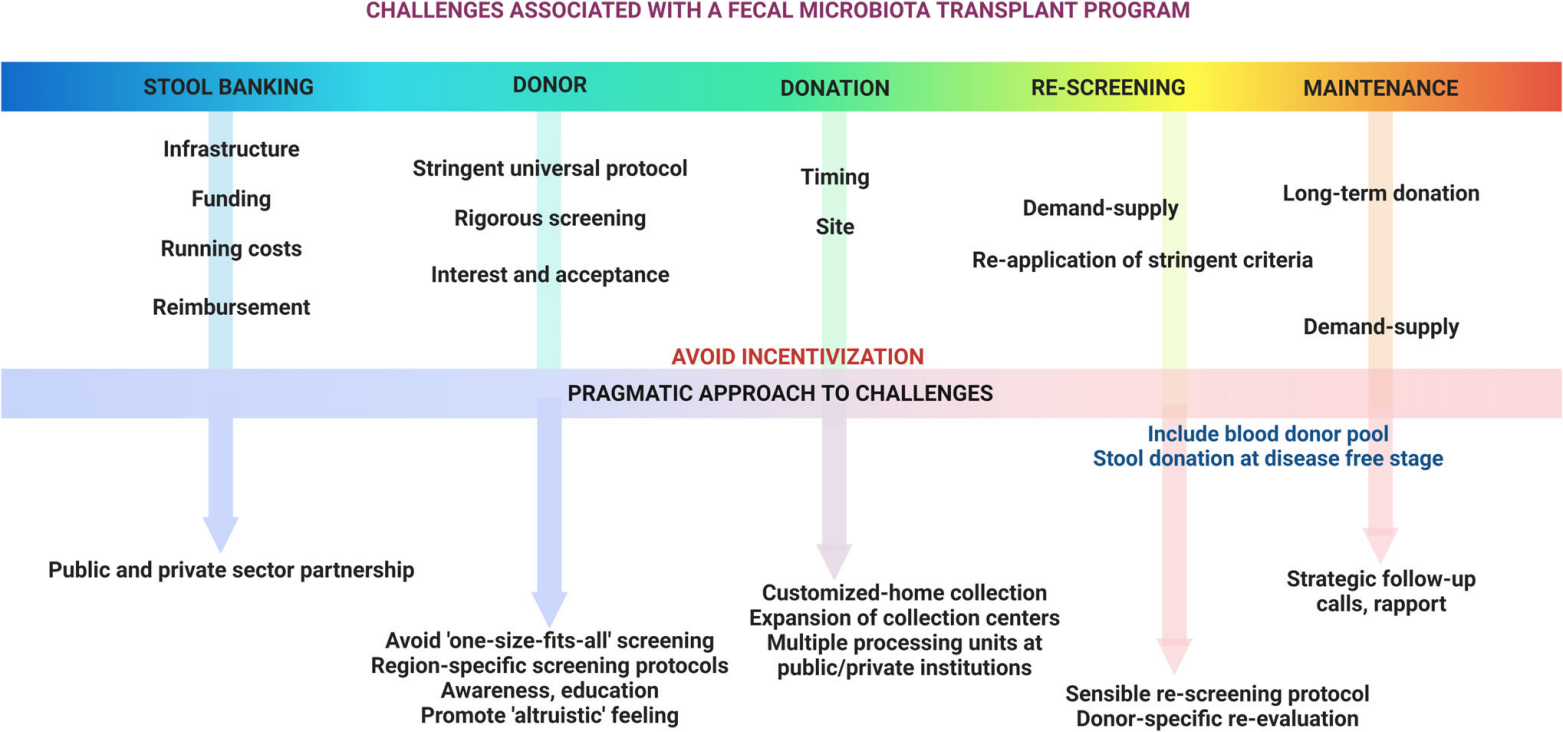
Challenges to stool donor recruitment, screening, stool banking, and maintenance of the demand–supply chain for fecal microbiota transplantation.

The study by Emily Tucker et al., like similar studies that came before it, highlights the persistent challenges faced by stool banking programs worldwide in the context of “healthy donor” enrolment, maintaining sustained donation, preventing donor attrition, and catering to FMT programs in clinical and research situations (Fig. 1).^7^ Incentivization of stool donations must be avoided to exclude unethical donations. To smoothen the edges, we must look at stool donation and processing protocols and banking programs from another angle. With improved public awareness and education on the importance of FMT, the embarrassment of donation and an improved sense of altruism towards donation can be addressed. Programs should be made “inclusive” rather than exclusion being the norm, considering the development and testing of pertinent screening protocols specific to regions rather than a “one‐size‐fits‐all” universal approach. A perfect example of such an approach is using prescreened donors from blood banking facilities for stool donation. Recruitment of stool donors from among blood donors in a Dutch cohort increased donor enrolment to 20%.^11^ Finally, the idea of stool banking and autologous FMT using the recipients' own stool samples screened and collected at a younger age when they are disease free can address future concerns of reduced supply when demand surges – and demand is sure to increase in the future – as the central role of microbial modulation for a host of disease conditions is slowly and steadily unlocked.^12^

In this regard, we must aim to “seek and find” rather than “search and destroy” potentially healthy stool donations in an inclusive protocol that is cohesive with the regulatory framework and donor willingness and continued acceptance.
